# IgG silencing induces apoptosis and suppresses proliferation, migration and invasion in LNCaP prostate cancer cells

**DOI:** 10.1186/s11658-016-0029-6

**Published:** 2016-12-03

**Authors:** Yawen Xu, Binshen Chen, Shaobo Zheng, Yong Wen, Abai Xu, Kai Xu, Bingkun Li, Chunxiao Liu

**Affiliations:** Department of Urology, Zhujiang Hospital, Southern Medical University, No. 253, Industrial Road, Guangzhou, Guangdong China

**Keywords:** LNCaP cells, Prostate cancer, RNA interference, Immunoglobulin G, Proliferation, Apoptosis, Migration, Invasion, Cell cycle, Caspase-3

## Abstract

Immunoglobulin G (IgG) has been implicated in the progression of various cancers. This study explored the role of IgG in the proliferation, apoptosis, cell cycle and in vitro invasive properties of LNCaP prostate cancer cells. We used IGHG1 small interfering RNA to silence IgG1 expression in LNCaP cells. The efficacy of IgG1 gene knockdown was confirmed using qPCR and western blotting. The colony formation, proliferation, migration and invasion abilities of LNCaP cells after transfection were assessed using colony-forming, flow cytometry and transwell assays. The expressions of PCNA and caspase-3 proteins in LNCaP cells after transfection were detected with immunofluorescence staining and western blotting. IgG1 silencing significantly decreased the colony formation, survival, cell cycle progression, migration and invasion of LNCaP cells (*p* < 0.05). IgG1 silencing also reduced the amount of the proliferation marker PCNA and induced formation of the apoptotic marker caspase-3 (*p* < 0.05). Our results show that IgG1 produced by LNCaP cells confers advantages for tumor cell survival, proliferation, migration and invasion, suggesting that IgG1 is a potential target for prostate cancer treatment.

## Introduction

Prostate cancer accounts for approximately 25% of new cancer diagnoses and is the second leading cause of cancer-related death in men [1, 2]. The incidence of prostate cancer varies by region and ethnicity. In China, prostate cancer is often diagnosed when patients have already reached advanced stages of the disease, so the tumors are not amenable to surgical resection [1, 3]. Often, the only recourse is systemic treatment that includes androgen deprivation therapy (targeting the androgen receptor pathway), chemotherapy, bone-directed therapy, radiation therapy or a combination of these treatments [4]. There is a high rate of recurrence and resistance to therapies often develops over time, so there is a strong need to identify additional novel molecular targets for alternative therapeutic strategies.

Immunoglobulins (Ig) are expressed by B lymphocytes and a variety of tumor tissues and cancer cell lines including breast, cervical, lung, hepatic, prostate and oral epithelial tumors [5–10]. Immunoglobulin G (IgG) accounts for 75–80% of the total Ig pool [11]. The role of IgG in cancer is a relatively new subject of study. The results implicate IgG in promoting cancer progression [12–14]. In lung cancer, breast cancer and esophageal squamous cell carcinomas, tumor-derived IgG not only enhances the growth and survival of cancer cells but also facilitates metastatic processes.

It is possible that IgG has pro-tumor effects due to its role in the immune system. For example, in papillary thyroid cancer, IgG co-localized with complement proteins, which may indicate the formation of immune complexes that allow the cancer cells to escape host immune responses [15]. However, high levels of endogenous IgG in the serum might also impair NK cell-mediated antibody-dependent cell cytotoxicity in antibody-based therapy [16, 17].

Alternative roles of IgG that are not related to immune function should also be considered in cancer cells. To date, relatively little is known about the role of prostate cancer-derived Ig. In prostate cancer patients, the serum levels of IgG, IgA or IgM are usually elevated [18, 19]. However, whether IgG exists intracellularly in tumor cells is a controversial question [10, 20]. Chen et al. found IgG heavy and light chain proteins localized in the cytoplasm and at the membrane of prostate cancer cells using the PC3 cell line [5]. They also documented expression of recombination activating gene 1 (RAG1) and RAG2. Consistent with their findings, we also found IgG expression in PC3 cells and in another prostate cancer cell line, LNCaP, in an earlier study [21].

LNCaP cells are hormone-dependent cancer cells that are typical of early-stage prostate cancer. This study aimed to address the role of IgG in prostate cancer using LNCaP cells. The method involved silencing IgG expression using RNA interference (RNAi) technology. Tumor cell survival advantages are conferred by a variety of mechanisms, including increased cell proliferation, cell cycle dysregulation and apoptosis resistance. Therefore, we assessed the effects of IgG knockdown on each of these outcomes, and on the migration and invasion abilities of LNCaP prostate cancer cells.

## Materials and methods

### Cell culture

Human androgen-dependent prostate cancer cells from the LNCaP line were obtained from Landbiology. The cells were maintained in Dublecco’s modified Eagle’s medium (DMEM; Hyclone) supplemented with 10% fetal bovine serum (FBS; Hyclone) at 37 °C in a humidified 5% CO_2_ atmosphere. Cells were used in the logarithmic growth phase.

### Transfection

The siRNA oligoribonucleotides were designed and synthesized by RiboBio. The siRNAs targeting human IgG1 mRNA were termed siIgG. siRNA that was not homologous to any human genome sequences was used as the negative control and termed siCon. The siIgG sequence targeted the constant region of the IgG1 heavy chain (target sequence: CTACACATCAGCAGGAATA). The sequences of the sense and anti-sense strands were 5′-CUACACAUCAGCAGGAAUA and 3′-dTdTGAUGUGUAGUCGUCCUUAU, respectively. The day prior to transfection, LNCaP cells were seeded on 6-well plates (5.0 × 10^5^ cells/well) or 96-well plates (1.0 × 10^4^ cells/well). The cells were allowed to grow until they were 70–80% confluent and then transfected with duplex siRNA using Lipofectamine 2000 (Invitrogen) according to the manufacturer’s instructions. The transfection efficiency was assessed via quantitative PCR (qPCR) 36 h after transfection.

### RNA isolation and qPCR

Total RNA was extracted using TRIzol (Invitrogen). cDNA was synthesized from 1 μg of RNA using the M-MLV reverse transcriptase system (Promega) according to the manufacturer’s instructions. The qPCR reaction contained 5 μl cDNA (1:20), 0.5 μl forward primer, 0.5 μM reverse primer, 10 μl 2× SYBR Green qPCR SuperMix (PE Applied Biosystems) and 4 μl ddH_2_O. qPCR was performed using an ABI Prism 7500 Sequence Detection System (Applied Biosystems). The reaction conditions were 95 °C for 5 min followed by 40 cycles consisting of 95 °C for 15 s, 60 °C for 15 s and 72 °C for 32 s. The sequences of the IGHG1 primers were: forward 5′-GCAGCCGGAGAACAACTACA-3′ and reverse 5′-TGGTTGTGCAGAGCCTCATG-3′. 18SrRNA was used as the internal reference (forward primer: 5′-CCTGGATACCGCAGCTAGGA-3′, reverse primer: 5′-GCGGCGCAATACGAATGCCCC-3′). Relative quantitation was performed using the ΔΔCt method.

### Western blotting

Total cell lysates were prepared and then separated using SDS-PAGE and transferred to a PVDF membrane (Millipore). The membranes were then blocked with 5% skim milk at room temperature for 1 h. The membranes were washed and incubated with primary antibody at 37 °C for 2 h and secondary antibody at 37 °C for 1 h. The primary antibodies used were: rabbit anti-human IgG (Dako) and horseradish peroxidase-conjugated anti-GAPDH (KangChen Bio-tech). The secondary antibodies used were: horseradish peroxidase-conjugated goat anti-rabbit and anti-mouse IgG (Dako). After washing, the proteins were visualized using the SuperSignal West Pico Chemiluminescent Substrate Kit (Pierce Biotechnology) according to the manufacturer’s instructions. Densitometry analysis was performed using AlphaImager 2200 analysis software.

### Colony-forming assay

Three groups of cells were plated in triplicate at 100 cells per well in 96-well plates. One the cells had formed colonies, they were stained with crystal violet and then photographed and counted. Experiments were independently repeated three times and the results were quantified as colony-forming efficiency (CFE) and surviving fraction (SF) using the equations:

$$ \mathrm{C}\mathrm{F}\mathrm{E} = \mathrm{number}\ \mathrm{of}\ \mathrm{colonies}/\mathrm{number}\ \mathrm{of}\ \mathrm{inoculated}\ \mathrm{cells} \times 100\% $$

$$ \mathrm{S}\mathrm{F} = \mathrm{number}\ \mathrm{of}\ \mathrm{experimental}\ \mathrm{group}\ \mathrm{colonies}/\mathrm{number}\ \mathrm{of}\ \mathrm{control}\ \mathrm{group}\ \mathrm{colonies} \times 100\% $$

### Cell cycle analysis

Cells were collected 48 h after transfection and fixed with 70% ethanol at 4 °C overnight. The cells were then stained with a cell cycle detection kit (KeyGen Bio-tech). Briefly, after one washing with PBS, the fixed cells were re-suspended in 500 μl PBS containing 50 μg/ml propidium iodide (PI), 100 μg/ml RNase A and 0.2% Triton X-100 and incubated at 4 °C in the dark for 30 min. Cells were analyzed using flow cytometry (BD Biosciences) using the ModFit software.

### Cell migration and invasion assays

For the migration assays, 10^5^ cells in 100 μl of serum-free medium were placed into the upper chamber of a transwell insert (BD Biosciences). For the invasion assays, 10^5^ cells in 100 μl serum-free medium were placed into the upper chamber of a transwell insert coated with Matrigel (BD Biosciences). Medium supplemented with 10% FBS (600 μl) was added to the lower chamber. The transwell plates were incubated at 37 °C in 5% CO_2_ for 12–48 h. The cells remaining on the upper membrane were removed with cotton wool. The cells that had migrated or invaded through the membrane were fixed with 4% paraformaldehyde, stained with crystal violet for 10 min, imaged and counted. Experiments were independently repeated three times.

### Immunofluorescence staining

Cells were grown on slides, transfected with siIgG or siCon, and 48 h later, fixed with 4% paraformaldehyde for 30 min at room temperature. After washing, the fixed cells were permeabilized with 0.2% Triton-X 100 for 5 min at room temperature, then blocked with 10% normal goat serum for 30 min, and incubated with primary antibodies against proliferating cell nuclear antigen (PCNA; Cell Signaling Technology) and caspase-3 (Abcam) at 4 °C overnight. The following day, the slides were incubated with fluorescently labeled secondary antibody (1:200; Life Technologies) in the dark for 1 h and 4′,6-diamidino-2-phenylindole (DAPI) for 5 min. Slides were mounted with antifade mounting medium (Beyotime). The slides were imaged and analyzed using Image-Pro Plus software (Media Cybernetics).

### Statistical analysis

All data are expressed as means ± standard deviation and reflect at least three independent experiments. One-way ANOVA and Tukey’s post-hoc test were used to compare the means of the three groups (LNCaP, siCon and siIgG). A p-value less than 0.05 was considered significant. All analyses were done with IBM SPSS Version 20 (SPSS Statistics V20, IBM Corporation).

## Results

### IgG1 knockdown inhibited the attachment-independent growth of LNCaP cells

**Fig. 1:**
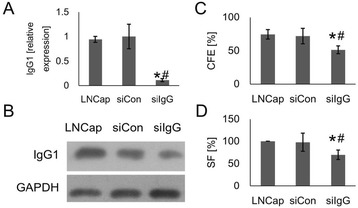
IGHG1 silencing suppresses the clonogenicity and survival of LNCaP cells. Cells were transfected with IgG1 (siIgG) and control siRNA (siCon). **a** – Cells were harvested at 36 h for qPCR to determine the mRNA expression levels. **b** – Cells were harvested at 48 h for western blotting to determine the protein expression levels. **c** – Transfected cells were used for a colony-forming assay. Colonies were stained with crystal violet and then counted to calculate the colony-forming efficiency (CFE). **d** – The same stained colonies were counted to determine the surviving fraction (SF). *p < 0.01 vs. siCon; ^#^ *p* < 0.01 vs. LNCaP (non-transfected control)

To investigate the role of IgG, LNCaP cells were transfected with siCon or siIgG to knockdown IgG1 expression. LNCaP cells that were not transfected with any siRNA were used as a second control group (LNCaP). The reduction in IgG1 was confirmed at the mRNA level via qPCR (Fig. 1a) and at the protein level via western blotting (Fig. 1b). IgG1 mRNA expression in the siIgG group (0.11 ± 0.03) was significantly (*p* < 0.01) lower than the expression in either the siCon (1 ± 0.25) or LNCaP (0.94 ± 0.06) groups. Similarly, IgG1 protein expression in the siIgG group (1.02 ± 0.09) was significantly (*p* < 0.01) lower than the expression in either the siCon (3.03 ± 0.11) or LNCaP (2.9 ± 0.21) groups. The IgG1 mRNA and protein expression levels in the siCon and LNCaP groups were not significantly (*p* > 0.05) different.

The effects of siRNA-mediated downregulation of IgG1 on the ability of LNCaP cells to form attachment-independent colonies (Fig. 1c) and on survival (Fig. 1d) were analyzed with colony-forming assays. The colony-forming efficiency (CFE) and surviving fraction (SF) of the siIgG group were significantly (*p* < 0.05) lower than the values for either of the two control groups 48 h after transfection.

These results indicated that the siRNA approach successfully reduced the expression of IgG1 at the mRNA and protein levels, and that reduced IgG1 expression impaired the ability of LNCaP cells to survive and form colonies.

### IgG1 knockdown inhibited cell cycle progression in LNCaP cells

**Fig. 2:**
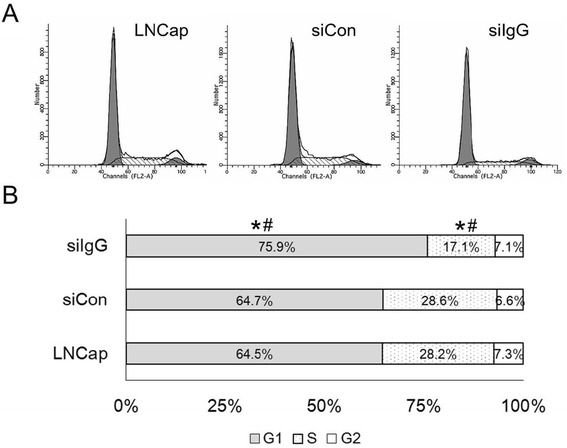
IGHG1 silencing attenuates the cell cycle progression of LNCaP cells. Cells were transfected with IgG1 (siIgG) and control siRNA (siCon) for 48 h, then harvested, fixed and stained with propidium iodide (PI). The cell cycle was analyzed by flow cytometry. **a** – Representative histograms. **b** – Quantitative results from three independent experiments. *p < 0.05 vs. siCon; ^#^ *p* < 0.05 vs. LNCaP (non-transfected control)

The effects of siRNA-mediated downregulation of IgG1 on cell cycle progression were examined via flow cytometry 48 h after transfection in cells stained with PI (Fig. 2a). Quantitative results are shown in Fig. 2b. The percentage of cells in the G1 phase in the siIgG group (75.88 ± 0.55) was significantly (*p* < 0.05) higher than the percentage for either the siCon (64.74 ± 0.29) or LNCaP (64.53 ± 0.35) groups. Concurrently, the percentage of cells in the S phase in the siIgG group (17.06 ± 0.35) was significantly (*p* < 0.05) lower than either the siCon (28.63 ± 2.09) or LNCaP (28.17 ± 0.17) groups, indicating that IgG1 knockdown blocked cell cycle progression.

### IgG1 knockdown inhibited migration and invasion of LNCaP cells

**Fig. 3:**
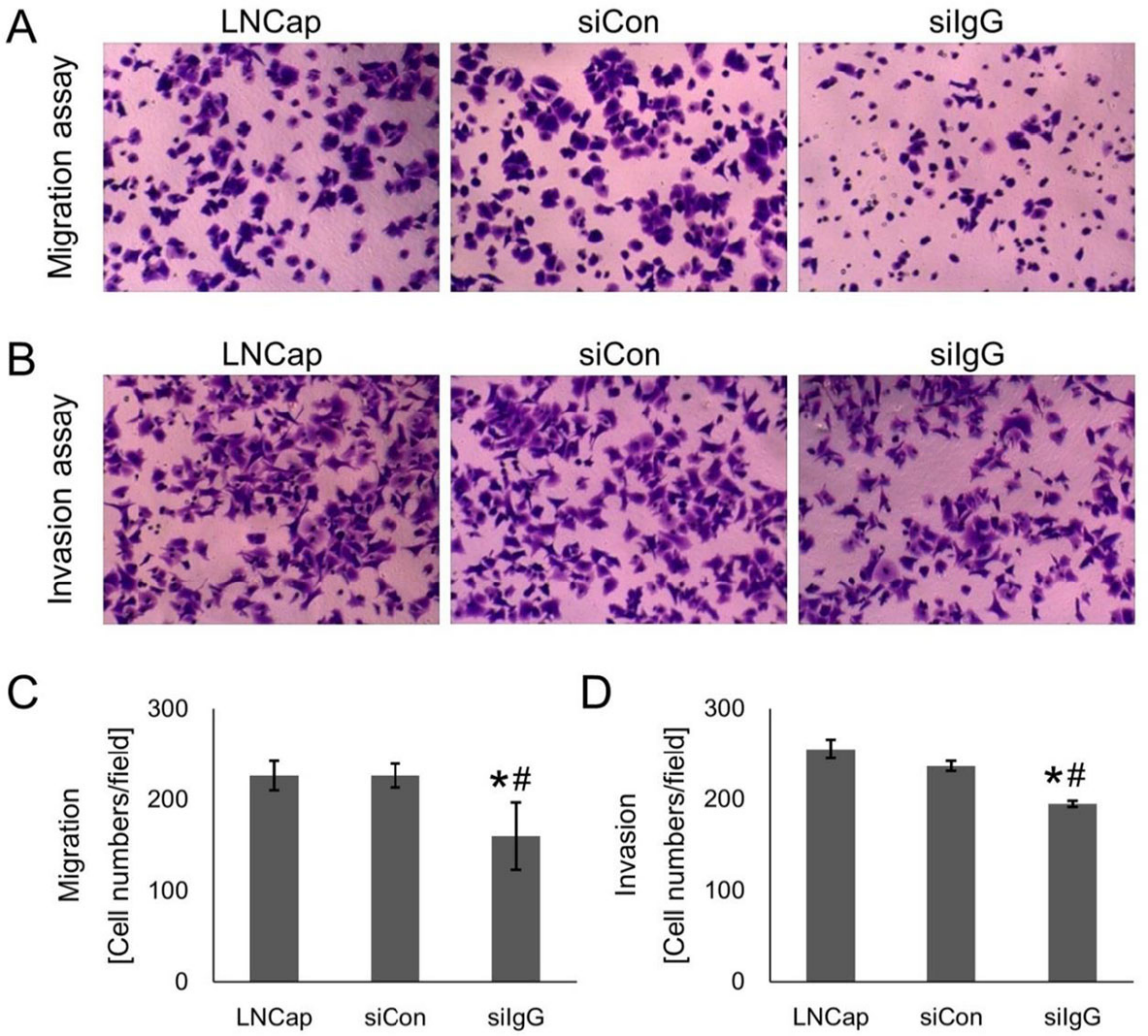
IGHG1 silencing reduces in vitro migration and invasion of LNCaP cells. Cells were transfected with IgG1 (siIgG) and control siRNA (siCon) and then seeded into transwells to determine **a** – The cell migration ability and **b** – The cell invasion ability. For migration assays, the cells were seeded in inserts containing fresh medium. For invasion assays, the inserts contained matrigel. Representative pictures are shown (scale bar, 100 μM). **c**, **d** – Quantitative results from three independent experiments are shown. *p < 0.05 vs. siCon; ^#^ *p* < 0.05 vs. LNCaP (non-transfected control)

Transwell experiments were performed to further investigate whether IgG1 knockdown affected the ability of LNCaP cells to migrate and invade. Representative images showing the number of cells migrating through the transwell (Fig. 3a) and invading the Matrigel substrate (Fig. 3b) are shown. Quantitative results indicated that the number of cells with the ability to migrate (Fig. 3c) and invade (Fig. 3d) was significantly (*p* < 0.05) reduced in the siIgG group compared to the two control groups. Thus, it is likely that IgG1 expression increases the migratory and invasive potential of LNCaP cells.

### IgG1 knockdown inhibited proliferation and induced apoptosis in LNCaP cells

**Fig. 4:**
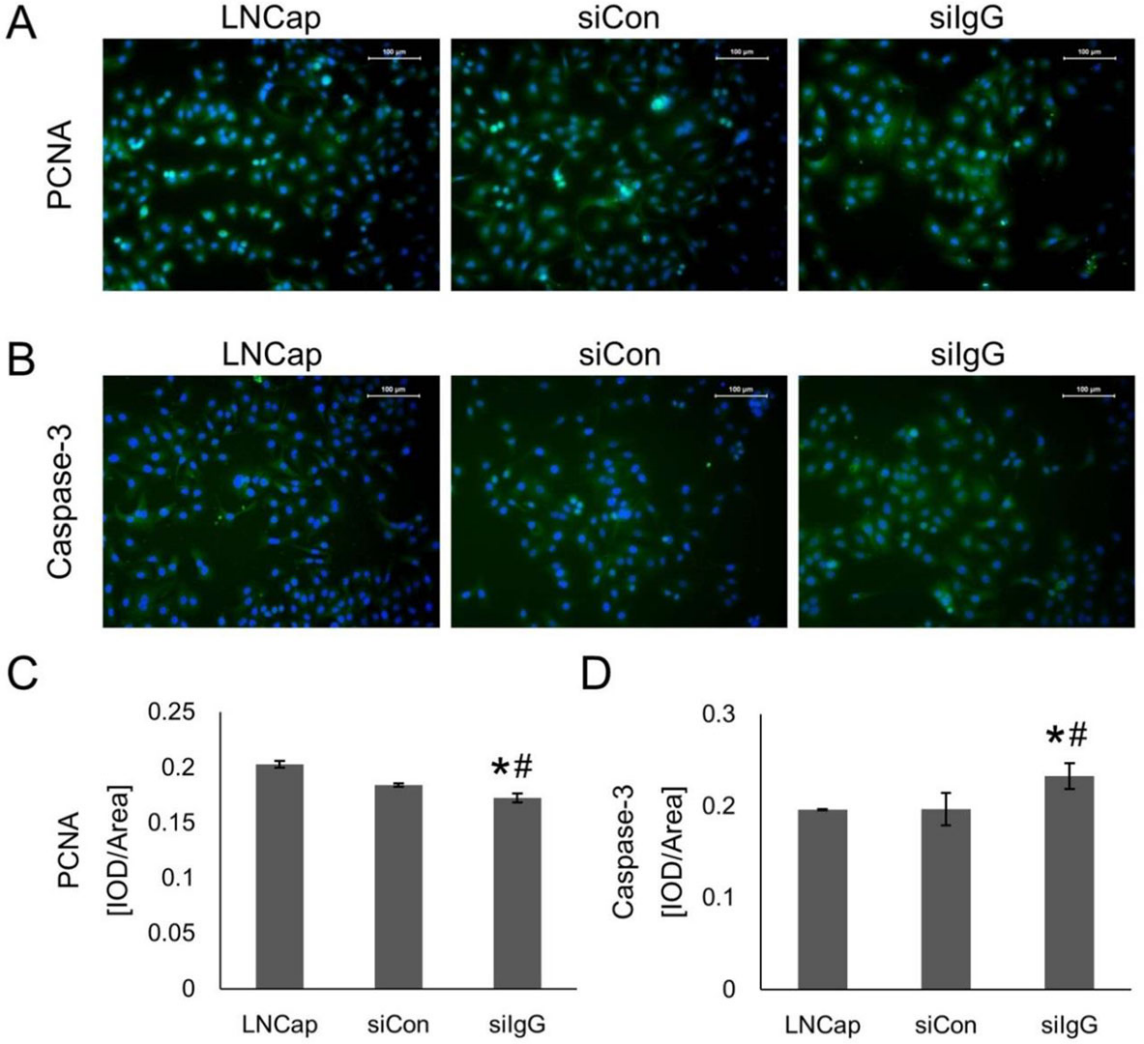
IGHG1 silencing decreases PCNA and increases caspase-3 in LNCaP cells. Cells were transfected with IgG1 (siIgG) and control siRNA (siCon) for 48 h and then fixed with 4% paraformaldehyde and stained for **a** – PCNA proliferation marker and **b** – caspase-3 apoptotic marker. Representative images are shown (scale bar, 100 μM). **c**, **d** – The integrated optical density (IOD) of green fluorescence and the area were quantified. *p < 0.05 vs. siCon; ^#^ *p* < 0.05 vs. LNCaP (non-transfected control)

Next, we investigated the effects of siRNA-mediated IgG1 downregulation on cell proliferation and apoptosis. Proliferation was assessed via immunofluorescence staining for the PCNA antigen in the siIgG, siCon and LNCaP groups. Figure 4a presents representative images showing the number of PCNA-positive cells in the siIgG group compared to the control groups. Quantitative analyses indicated that the number of PCNA-positive cells was significantly (*p* < 0.05) lower in the siIgG group than in either control group (Fig. 4c). The percentage of apoptotic cells was assessed using the marker caspase-3. Representative images of caspase-3 staining are shown in Fig. 4b. The number of caspase-3 positive cells was significantly (*p* < 0.05) higher in the siIgG group than in the two control groups (Fig. 4d). These effects are consistent with the tumor suppressive effects of IgG1 knockdown.

## Discussion

Despite the progress in developing new treatments, the mortality of late-stage prostate cancer remains high, meaning there is still a need to investigate novel therapeutics. We and other researchers have found that IgG is expressed in prostate cancer cell lines and prostate cancer tissues [21, 22]. This study was undertaken to understand the role of IgG expression in the prostate cancer cell line LNCaP using siRNA to knockdown IgG1. Silencing IgG increased apoptosis and inhibited proliferation, cell migration and invasion.

The mechanisms conferring tumor cell survival advantages are a complex network involving signaling of cell proliferation, cell cycle and apoptosis [23]. Our study found that silencing IgG in LNCaP cells exerts multiple regulation forces on the network. We found that cell proliferation was inhibited with IgG siRNA through the downregulation of proliferating cell nuclear antigen (PCNA), which is an auxiliary factor for DNA polymerase δ in eukaryotic cells and is essential for DNA replication [24]. Caspase-3 is the main executor of the caspase cascade in apoptotic cells. It was previously shown that pHyde, a novel gene identified in Dunning rat prostate cancer cells, induces apoptosis in a dose-dependent manner by activating caspase-3 [25]. It is possible that the IgG1-mediated inhibition of apoptosis involves pHyde in LNCaP cells, but that remains to be confirmed. An additional report demonstrated that the loss of caspase-1 is potentially a step toward aberrant apoptosis during prostate tumorigenesis [26]. Our results suggest that silencing IgG1 may be a novel approach to increasing caspase-3, and possibly caspase-1, to correct the loss of apoptosis in prostate cancer.

In addition to its pro-apoptotic effects, silencing IgG1 inhibited cell proliferation. Although cancer cells have been found to express IgG, the molecular mechanisms by which tumor-derived IgG promotes tumor growth are unclear. Several hypotheses have been proposed.

Wang et al. found that tumor-derived IgG promotes tumor cell growth and proliferation by inducing the production of reactive oxygen species (ROS) [27]. They identified that oxidative stress-related proteins, such as receptor of activated protein kinase C 1 (RACK1), ras-related nuclear protein (RAN) and peroxiredoxin 1 (PRDX1), were IgG-interacting proteins in HeLa cells (cervical cancer). ROS scavengers inhibited the growth of IgG-deficient cancer cells by suppressing the mitogen-activated protein kinase/extracellular-regulated kinase (MAPK/ERK) pathway.

Alternatively, a recent study demonstrated that cancer-derived IgG promotes cervical cancer cell proliferation by enhancing pro-inflammatory cytokine production in response to lipopolysaccharide (LPS) binding to Toll-like receptor 4 (TLR4) [28]. Knockdown of IgG suppressed NF-κB and MAPK phosphorylation, which in turn impaired NF-κB nuclear translocation and the activity of NF-κB responsive elements. These data suggest that IgG may be a novel therapeutic target for treating inflammation-mediated cancers, including prostate cancer [29], and that tumor-derived IgG may play a similar proinflammatory role in prostate cancer.

The pro-tumor effects of IgG may also include a role in metatstatic cancer. We found that both cell migration and invasion were reduced when IgG1 was knocked down. Jiang et al. also found that knockdown of IgG decreases the proliferation, migration and attachment of lung cancer cells in vitro, and that IgG expression correlated with metastasis-associated gene 1 (MTA1) expression and lymph node metastases in vivo [12]. In a breast cancer model, IgG purified from the blood of breast cancer patients stimulated muscarinic acetylcholine receptors to regulate cell migration and metalloproteinase-9 (MMP-9) activity in the breast cancer cell line MCF7 [30]. The molecular mechanisms might involve the phospholipase C, nitric oxide synthase and/or protein kinase C pathways. It is not clear whether any of the pathways described are associated with the pro-tumor functions of IgG in prostate cancer.

The Ig constant regions of human IgA, IgM, IgD, IgG3, IgG1 and IgE can be detected in many cancer cell lines via nested RT-PCR [7]. However, the individual functions of each Ig isotype in cancers are not clearly identified. Zheng et al. showed that the cancer cell-derived Ig alpha heavy chain promotes access to S phase and the growth of cancer cells [31]. Yang et al. examined the role of expression of Ig light chain, Ig kappa and Ig lambda in colorectal cancer cells [32]. They found that Ig kappa and lambda help to stabilize the anti-apoptotic protein Bcl-xL. Knockdown of the genes of Ig kappa and/or Ig lambda suppresses Bcl-xL expression and induces apoptosis. The roles of other Ig isotypes in prostate cancer remain to be addressed, as does whether IgG silencing altered apoptosis in LNCaP cells by downregulating Bcl-xL.

In summary, knockdown of IgG expression by RNA interference inhibited cell proliferation and increased apoptosis in a human prostate cancer LNCaP cell line. While these results have implications for the role of IgG in prostate cancer progression, they are limited to one cell line and one siRNA, and must be confirmed in other systems. The results suggest that IgG is a potential target for a novel therapeutic strategy for prostate cancer.

## Abbreviations

CFE: Colony-forming efficiency
DAPI: 4′,6-diamidino-2-phenylindole
IgG: Immunoglobulin G
PCNA: Proliferating cell nuclear antigen
PI: Propidium iodide
RAG1: Recombination activating gene 1
RNAi: RNA interference

## References

[1] Siegel R, Naishadham D, Jemal A (2012). Cancer statistics, 2012. CA Cancer J Clin.

[2] Siegel RL, Miller KD, Jemal A (2015). Cancer statistics, 2015. CA Cancer J Clin.

[3] Yang L, Parkin DM, Whelan S, Zhang S, Chen Y, Lu F, Li L (2005). Statistics on cancer in China: cancer registration in 2002. Eur J Cancer Prev.

[4] DeSantis CE, Lin CC, Mariotto AB, Siegel RL, Stein KD, Kramer JL, Alteri R, Robbins AS, Jemal A (2014). Cancer treatment and survivorship statistics, 2014. CA Cancer J Clin.

[5] Chen Z, Gu J (2007). Immunoglobulin G expression in carcinomas and cancer cell lines. FASEB J.

[6] Chen Z, Qiu X, Gu J (2009). Immunoglobulin expression in non-lymphoid lineage and neoplastic cells. Am J Pathol.

[7] Kimoto Y (1998). Expression of heavy-chain constant region of immunoglobulin and T-cell receptor gene transcripts in human non-hematopoietic tumor cell lines. Gene Chromosome Canc.

[8] Zhu X, Li C, Sun X, Mao Y, Li G, Liu X, Zhang Y, Qiu X (2008). Immunoglobulin mRNA and protein expression in human oral epithelial tumor cells. Appl Immunohistochem Mol Morphol.

[9] Lei Y, Huang T, Su M, Luo J, Korteweg C, Li J, Chen Z, Qiu Y, Liu X, Yan M, Wang Y, Gu J (2014). Expression and distribution of immunoglobulin G in the normal liver, hepatocarcinoma and postpartial hepatectomy liver. Lab Invest.

[10] Qiu X, Zhu X, Zhang L, Mao Y, Zhang J, Hao P, Li G, Lv P, Li Z, Sun X, Wu L, Zheng J, Deng Y, Hou C, Tang P, Zhang S, Zhang Y (2003). Human epithelial cancers secrete immunoglobulin g with unidentified specificity to promote growth and survival of tumor cells. Cancer Res.

[11] Terry WD, Fahey JL (1964). Subclasses of Human Gamma-2-Globulin Based on Differences in the Heavy Polypeptide Chains. Science.

[12] Jiang C, Huang T, Wang Y, Huang G, Wan X, Gu J (2014). Immunoglobulin G expression in lung cancer and its effects on metastasis. Plos One.

[13] Ma C, Wang Y, Zhang G, Chen Z, Qiu Y, Li J, Luo J, Huang B, Jiang C, Huang G, Wan X, Korteweg C, Gu J (2013). Immunoglobulin G expression and its potential role in primary and metastatic breast cancers. Curr Mol Med.

[14] Zhang L, Hu S, Korteweg C, Chen Z, Qiu Y, Su M, Gu J (2012). Expression of immunoglobulin G in esophageal squamous cell carcinomas and its association with tumor grade and Ki67. Hum Pathol.

[15] Qiu Y, Korteweg C, Chen Z, Li J, Luo J, Huang G, Gu J (2012). Immunoglobulin G expression and its colocalization with complement proteins in papillary thyroid cancer. Mod Pathol.

[16] Nechansky A, Schuster M, Jost W, Siegl P, Wiederkum S, Gorr G, Kircheis R (2007). Compensation of endogenous IgG mediated inhibition of antibody-dependent cellular cytotoxicity by glyco-engineering of therapeutic antibodies. Mol Immunol.

[17] Preithner S, Elm S, Lippold S, Locher M, Wolf A, da Silva AJ, Baeuerle PA, Prang NS (2006). High concentrations of therapeutic IgG1 antibodies are needed to compensate for inhibition of antibody-dependent cellular cytotoxicity by excess endogenous immunoglobulin G. Mol Immunol.

[18] Beneduce L, Prayer-Galetti T, Giustinian AM, Gallotta A, Betto G, Pagano F, Fassina G (2007). Detection of prostate-specific antigen coupled to immunoglobulin M in prostate cancer patients. Cancer Detect Prev.

[19] Golda R, Wolski Z, Wyszomirska-Golda M, Madalinski K, Michalkiewicz J (2004). The presence and structure of circulating immune complexes in patients with prostate tumors. Med Sci Monit.

[20] Babbage G, Ottensmeier CH, Blaydes J, Stevenson FK, Sahota SS (2006). Immunoglobulin heavy chain locus events and expression of activation-induced cytidine deaminase in epithelial breast cancer cell lines. Cancer Res.

[21] Pan B, Zheng S, Liu C, Xu Y (2013). Suppression of IGHG1 gene expression by siRNA leads to growth inhibition and apoptosis induction in human prostate cancer cell. Mol Biol Rep.

[22] Liu Y, Chen Z, Niu N, Chang Q, Deng R, Korteweg C, Gu J (2012). IgG gene expression and its possible significance in prostate cancers. Prostate.

[23] Evan GI, Vousden KH (2001). Proliferation, cell cycle and apoptosis in cancer. Nature.

[24] Cappello F, Ribbene A, Campanella C, Czarnecka AM, Anzalone R, Bucchieri F, Palma A, Zummo G (2006). The value of immunohistochemical research on PCNA, p53 and heat shock proteins in prostate cancer management: a review. Eur J Histochem.

[25] Zhang X, Steiner MS, Rinaldy A, Lu Y (2001). Apoptosis induction in prostate cancer cells by a novel gene product, pHyde, involves caspase-3. Oncogene.

[26] Winter RN, Kramer A, Borkowski A, Kyprianou N (2001). Loss of caspase-1 and caspase-3 protein expression in human prostate cancer. Cancer Res.

[27] Wang J, Lin D, Peng H, Huang Y, Huang J, Gu J (2013). Cancer-derived immunoglobulin G promotes tumor cell growth and proliferation through inducing production of reactive oxygen species. Cell Death Dis.

[28] Wang J, Lin D, Peng H, Shao J, Gu J (2014). Cancer-derived immunoglobulin G promotes LPS-induced proinflammatory cytokine production via binding to TLR4 in cervical cancer cells. Oncotarget.

[29] Taverna G, Pedretti E, Di Caro G, Borroni EM, Marchesi F, Grizzi F (2015). Inflammation and prostate cancer: friends or foe?. Inflamm Res.

[30] Pelegrina LT, Lombardi MG, Fiszman GL, Azar ME, Morgado CC, Sales ME (2013). Immunoglobulin g from breast cancer patients regulates MCF-7 cells migration and MMP-9 activity by stimulating muscarinic acetylcholine receptors. J Clin Immunol.

[31] Zheng H, Li M, Liu H, Ren W, Hu DS, Shi Y, Tang M, Cao Y (2007). Immunoglobulin alpha heavy chain derived from human epithelial cancer cells promotes the access of S phase and growth of cancer cells. Cell Biol Int.

[32] Yang SB, Chen X, Wu BY, Wang MW, Cai CH, Cho DB, Chong J, Li P, Tang SG, Yang PC (2009). Immunoglobulin kappa and immunoglobulin lambda are required for expression of the anti-apoptotic molecule Bcl-xL in human colorectal cancer tissue. Scand J Gastroentero.

